# Nasal transmission of equine parvovirus hepatitis

**DOI:** 10.1111/jvim.16569

**Published:** 2022-10-17

**Authors:** Joy E. Tomlinson, Gerlinde R. Van de Walle

**Affiliations:** ^1^ Baker Institute for Animal Health, College of Veterinary Medicine Cornell University Ithaca New York USA

**Keywords:** biosecurity, intranasal, oral, Theiler's disease

## Abstract

**Background:**

Equine parvovirus hepatitis (EqPV‐H) is highly prevalent and causes subclinical to fatal hepatitis, which can occur in outbreaks. Whereas iatrogenic transmission is well documented, the mode of horizontal transmission is not known. The virus is shed in nasal, oral and fecal secretions, and PO transmission has been reported in a single horse.

**Hypothesis/Objective:**

Investigate the efficiency of PO and nasal transmission of EqPV‐H in a larger cohort.

**Methods:**

Prospective experimental transmission study. Eleven EqPV‐H‐negative horses were inoculated with 5 × 10^6^ genome equivalents EqPV‐H. Serum PCR and serology for EqPV‐H were performed weekly and monthly, respectively. Horses first were inoculated PO, and then intranasally 8 weeks later.

**Results:**

No horse became viremic or seroconverted within 8 weeks after PO inoculation. After intranasal inoculation, 5 horses became viremic within 6 to 12 weeks and seroconverted within 10 to 19 weeks. After a period without monitoring from 12 to 19 weeks postinoculation, another 5 horses were found to be viremic at 19 to 22 weeks. The second set of 5 horses could have been infected by horizontal transmission from the first 5 because of cohousing.

**Conclusions and Clinical Importance:**

We demonstrated that EqPV‐H can be transmitted nasally. The prolonged eclipse phase before detectable viremia indicates biosecurity measures to control spread could be impractical.

**Abbreviations**
AHDC: Animal Health Diagnostic Center
AST: aspartate aminotransferase
CK: creatine kinase
EqPV‐H: equine parvovirus‐hepatitis
GE: genome equivalents
GGT: gamma glutamyltransferase
GLDH: glutamate dehydrogenase
IN: intranasal
IV: intravenous
LIPS: luciferase immunoprecipitation system
PBS: phosphate‐buffered saline
PO: per os
qPCR: quantitative polymerase chain reaction
Ruc‐Ag: renilla luciferase‐VP1 antigen
SDH: sorbitol dehydrogenase
VP1: viral protein 1

## 1 INTRODUCTION

Equine parvovirus hepatitis (EqPV‐H) recently has been described as an important cause of viral hepatitis in horses. Experimental infection studies showed that the virus commonly causes subclinical to mild clinical hepatitis,^1^ and case series found that EqPV‐H is associated with Theiler's disease, also known as acute hepatic necrosis.^2^, ^3^ Equine parvovirus hepatitis infections and Theiler's disease, which is often fatal, commonly occur after treatment with equine origin biologic products such as tetanus antitoxin, botulinum antitoxin, or others.^2^, ^4^, ^5^, ^6^, ^7^, ^8^, ^9^, ^10^, ^11^, ^12^, ^13^ The incubation period to detectable viremia is on average between 1 and 6 weeks after iatrogenic transmission, and onset of subclinical or clinical hepatitis usually ranges between 4 and 10 weeks after administration of an equine origin biologic, although it can be as long as 14 weeks.^1^, ^6^, ^14^, ^15^ However, many cases of EqPV‐H infection and Theiler's disease occur without a history of treatment with an equine origin biologic product, and the disease can occur as outbreaks on individual farms, suggesting that alternative natural routes of horizontal transmission are likely.^3^

Equine parvovirus hepatitis is widely distributed around the world with a viremia prevalence of 4% to 20% and a seroprevalence of 15% to 35%,^16^, ^17^, ^18^, ^19^, ^20^, ^21^, ^22^, ^23^, ^24^, ^25^, ^26^ although the prevalence can vary widely across premises. On premises where at least 1 horse has developed Theiler's disease, over 50% of in‐contact horses typically are infected, suggesting efficient horizontal transmission.^3^, ^27^ Insect vectoring has been proposed based on (i) the observation that spontaneous cases of Theiler's disease show a seasonal distribution in the summer to autumn^3^ and (ii) the fact that other blood‐borne viruses of horses, such as equine infectious anemia virus, are mechanically transmitted by biting flies.^28^, ^29^ Our recently attempted experimental transmission via horse fly bites was unsuccessful, but this result does not rule out biting fly transmission because of the limited number of bites used per horse in that study.^1^ Transmission by inhalation or ingestion also has been proposed based on (i) the fact that these are common transmission routes for many parvoviruses^30^, ^31^, ^32^ and (ii) our recent finding that EqPV‐H is shed intermittently via oral, nasal, and fecal secretions for at least 10 weeks after experimental IV inoculation.^1^ Furthermore, because we identified potential PO transmission in 1 out of 2 inoculated horses,^1^ the goal of our present study was to perform PO and nasal inoculations with EqPV‐H in a larger number of horses to evaluate transmission efficiency by these routes. Indeed, transmission by inhalation or ingestion more easily would explain the widespread distribution and high prevalence of EqPV‐H among horses when compared to mechanical insect vectoring, which would be more restricted in seasonality and geography.

## 2 MATERIALS AND METHODS

### 2.1 Animals

Eleven EqPV‐H serum quantitative polymerase chain reaction (qPCR) negative and luciferase immunoprecipitation system (LIPS) seronegative horses were sourced from the teaching and research herd at Cornell University or from donations. All procedures were approved by Cornell University Institutional Animal Care and Use Committee IACUC #2014‐0024.

### 2.2 Inoculations and sampling

Unfasted horses were inoculated PO with 5 × 10^6^ genome equivalents (GE) EqPV‐H in equine serum, diluted in phosphate‐buffered saline (PBS) to a final volume of 5 mL. Eight weeks later, horses were inoculated intranasally (IN) with the same inoculum using a 6‐in. soft rubber catheter with diffuser tip. Horses that had not become viremic or seroconverted were challenged IV to document susceptibility to EqPV‐H infection. Serum EqPV‐H qPCR was performed weekly, and LIPS serology approximately monthly.

### 2.3 Quantitative polymerase chain reaction

Viral nucleic acids were extracted from serum with Qiagen Viral RNA Mini kit (catalog no. 52906) according to the manufacturer's instructions. No DNase treatment was applied. The PCR was performed using primers EqPV‐H q VP1 F15/R15 as previously described.^1^ All PCR reactions were run on the QuantStudio 3 and analyzed on the QuantStudio 3 software (ThermoFisher). Suspect positive samples near the limit of detection were confirmed by submission to the New York State Animal Health Diagnostic Center (AHDC), which uses the PCR method described previously.^2^

### 2.4 Serum biochemistry and hematology

Serum biochemical analysis and CBCs were performed by the AHDC on a fee‐for‐service basis. Biochemical tests performed were aspartate aminotransferase (AST), sorbitol dehydrogenase (SDH), glutamate dehydrogenase (GLDH), gamma glutamyltransferase (GGT), and creatine kinase (CK) activities, and bile acid, total, direct, and indirect bilirubin, and triglyceride concentrations. Reference intervals were AST, 222 to 489 U/L; SDH, 1 to 6 U/L; GLDH, 2 to 10 U/L; GGT, 8 to 33 U/L; creatine kinase, 171 to 567 U/L; bile acids, 2 to 10 μmol/L; total bilirubin, 0.5 to 2.1 mg/dL; direct bilirubin, 0.1 to 0.3 mg/dL; indirect bilirubin, 0.3 to 2.0 mg/dL; and triglycerides, 14 to 65 mg/dL.

Hemogram parameters and reference intervals were: hematocrit, 34% to 46%; hemoglobin, 11.8 to 15.9 g/dL; red blood cell count, 6.6 to 9.7 × 10^6^/μL; mean corpuscular volume, 43 to 55 fL; mean corpuscular hemoglobin, 15 to 20 pg; mean corpuscular hemoglobin concentration 34 to 37 g/dL; red cell distribution width, 16.3% to 19.3%; nucleated red blood cells, 0/100 white blood cells; white blood cells, 5.2 to 10.1 × 10^3^/μL; segmented neutrophils, 2.7 to 6.6 × 10^3^/μL; band neutrophils 0.0 to 0.1 × 10^3^/μL; lymphocytes, 1.2 to 4.9 × 10^3^/μL; monocytes, 0.0 to 0.6 × 10^3^/μL; eosinophils, 0.0 to 1.2 × 10^3^/μL; basophils, 0.0 to 0.2 × 10^3^/μL; platelet count, 94 to 232 × 10^3^/μL; mean platelet volume, 5.3 to 8.4 fL; and total protein, 5.2 to 7.8 g/dL. Blood smears were examined manually to confirm automated results.

### 2.5 Serology

Antiviral protein 1 (VP1) immunoglobulin G antibodies were detected in serum by LIPS assay, as previously described,^16^ with the following modifications. Briefly, the pREN2‐EqPV‐H plasmid (a kind gift from Dr. Peter Burbelo, National Institutes of Health, Washington DC) was transformed into TOP10 *Escherichia coli*, which were transfected into Cos1 cells. The renilla luciferase‐VP1 antigen (Ruc‐Ag) was harvested as a cell lysate using a renilla luciferase assay system lysis buffer (Promega). Serum samples, tested in duplicate, were diluted in buffer A (50 mM Tris, pH 7.5, 100 mM NaCl, 5 mM MgCl_2_, 1% Triton X‐100), incubated with Ruc‐Ag for 1 h, followed by incubation with protein A/G beads for 1 h in a 96‐well v‐bottom plate on a rotary platform. For washing, consisting of 6 washes with 200 μL buffer A and 1 wash with 200 μL PBS, beads were pelleted at 2000*g* for 3 min and supernatants discarded. Beads were resuspended in 10 μL PBS and transferred to a white flat bottom 96‐well plate for luminescence reading in an Infinite M‐plex microplate reader (Tecan). Controls included EqPV‐H seropositive and seronegative horse serum samples, and the cutoff was set at the average of all preinoculation sera +4 standard deviations of preinoculation sera.

### 2.6 Statistics

Descriptive statistics were performed.

## 3 RESULTS

### 3.1 Overview experimental inoculation study

**FIGURE 1:**
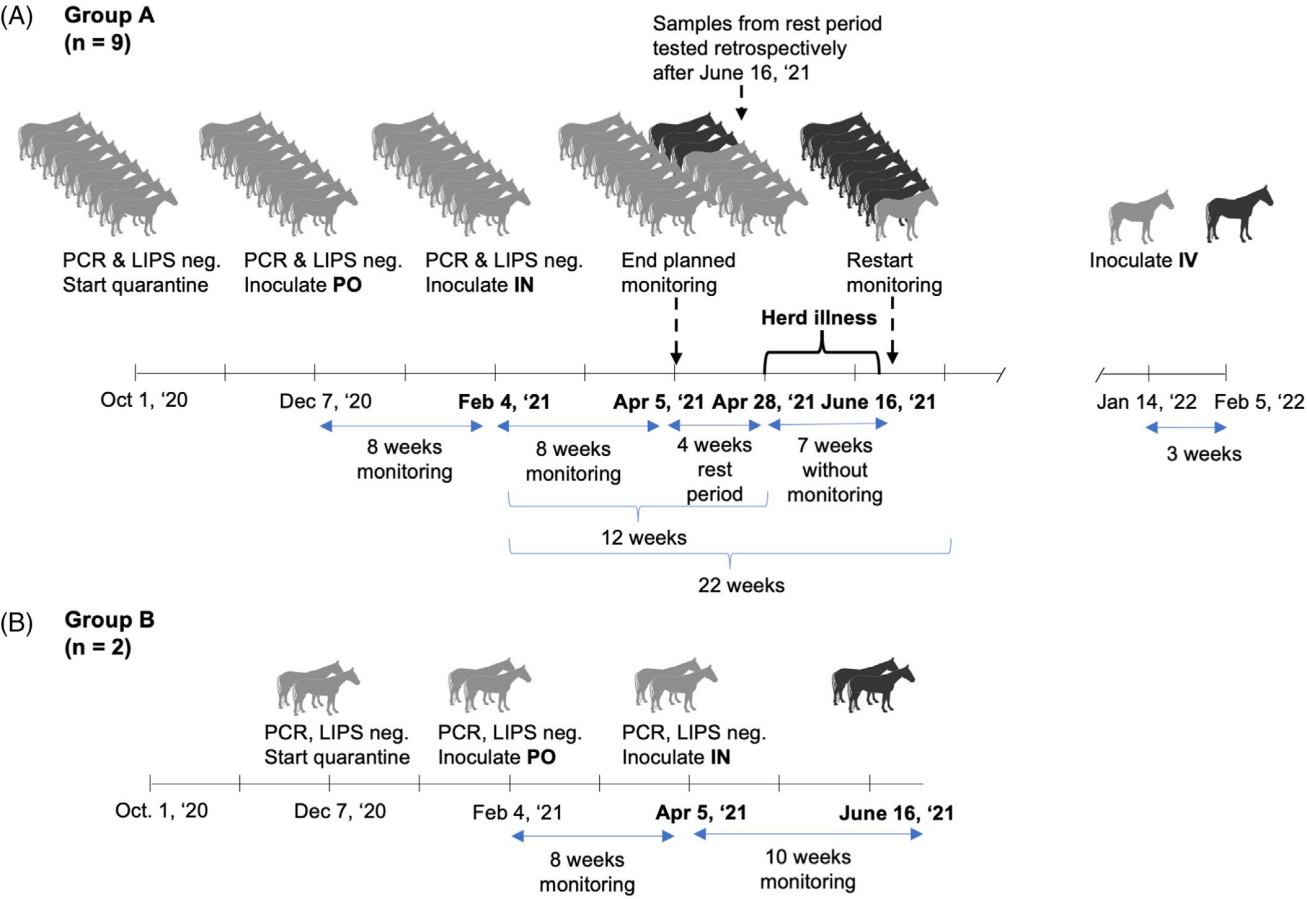
Study timeline. Horses were enrolled in 2 groups: group A, 9 horses; group B, 2 horses. Before inoculations, horses were screened by physical examination, serum biochemistry, CBC, serum qPCR, and LIPS serology. Horses were quarantined for 8 weeks, and then were inoculated with 5 × 10^6^ genome equivalents (GE) EqPV‐H in horse serum PO. They were inoculated IN 8 weeks later, and monitored an additional 8 weeks. (A) In group A, horses were given routine veterinary care 8 weeks after IN inoculation, followed by a 4‐week rest period. Then, 12 weeks after IN inoculation, the herd developed an upper respiratory tract infection and monitoring was suspended for 7 weeks. Weekly monitoring was restarted at week 19, after horses clinically recovered. Susceptibility to EqPV‐H infection was later confirmed by IV inoculation of the single horse that had not been infected. (B) In group B, both horses became infected within 10 weeks after IN inoculation. Gray horse, EqPV‐H qPCR negative; black horse, EqPV‐H qPCR positive

We performed an experimental inoculation study using 11 horses that were 9 mares and 2 geldings between the ages of 3 and 14 years old and of various breeds (9 Thoroughbred, 1 Quarter Horse, and 1 Appendix Quarter Horse). All horses were healthy by physical examination, CBC, and serum biochemistry, and confirmed to be EqPV‐H negative by serum qPCR and LIPS serology at the start of their 8‐week quarantine and again at the start of the inoculation study. Nine horses (group A) were enrolled and housed together, and an additional 2 horses (group B; horses MA and SIG) were enrolled later and housed together (Figure 1).

### 3.2 Group A inoculations and results

Group A (n = 9) horses were inoculated first PO with 5 × 10^6^ GE EqPV‐H in equine serum, diluted in PBS to a final volume of 5 mL. Serum EqPV‐H qPCR was performed weekly for 8 weeks and LIPS serology at 0 and 8 weeks. None of the horses became viremic and all were seronegative 8 weeks after PO inoculation (February 4th; Figure 1A).

All 9 horses then were inoculated IN with the same inoculum as described above, using a 6‐in. soft rubber catheter with diffuser tip, and their heads were held elevated for approximately 30 seconds after inoculation. Again, serum EqPV‐H qPCR was performed weekly for 8 weeks and LIPS serology approximately monthly. None of the 9 horses from group A became viremic by the end of the 8‐week monitoring period after IN inoculation (April 5th; Figure 1A). Intravenous inoculation was planned as the next step to demonstrate the horses were susceptible to EqPV‐H.

### 3.3 Rest period

The 8‐week monitoring post‐IN inoculation was followed by an 11‐week rest period (Figure 1A). Samples were collected between weeks 8 and 12, but testing was not performed in real‐time. No samples were obtained in weeks 13 to 18 after IN inoculation. This initial 4‐week rest period was implemented to allow the immune response to return to baseline^33^, ^34^ after administering spring vaccines and dewormer, and before initiating IV inoculation to demonstrate susceptibility to EqPV‐H, as previously described.^1^

At the end of the planned 4‐week rest period (April 28th) upper respiratory infection was detected in the herd (Figure 1A). Horses were variably affected with mucoid to mucopurulent nasal discharge and coughing, and 1 horse (Horse RK) developed fever up to 104.5°F. Nasal swabs from 3 horses (Horses RK, HA, and FO) were submitted to AHDC and were qPCR negative for all tested equine upper respiratory infectious agents (equine adenovirus 1 and 2, equine arteritis virus, equine herpesvirus 1 and 4, equine rhinitis virus A and B, influenza virus, streptococcus equine subsp. equi) and EqPV‐H. Horses were treated according to veterinary guidance, and all recovered fully. The study was suspended until 1 month after resolution of clinical signs to allow the immune response to return to baseline.

### 3.4 Continued monitoring after rest period

On June 16th, 19 weeks after IN inoculation, all horses from group A were re‐enrolled for planned IV inoculations. Retrospective analysis of the week 9 to 12 (April 5th to 28th) samples indicated that 3 horses had developed viremia by week 12. Horse RQ was viremic around week 10, and Horses BU and FO around week 12 after IN inoculation (Figure 1A).

**FIGURE 2:**
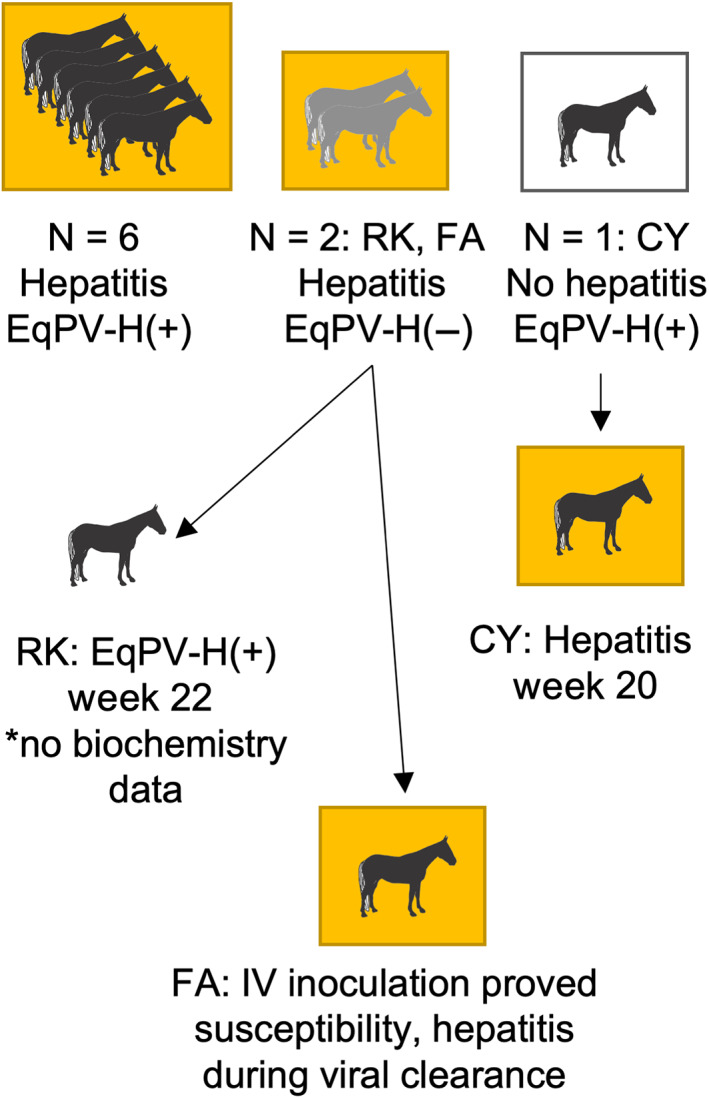
Biochemistry and serum EqPV‐H qPCR results of 9 horses in group A at week 19 after intranasal inoculation. Yellow background, horses had hepatitis as detected by increases in serum GGT, SDH, GLDH, AST, or some combination of these with normal CK activity. Gray horse, EqPV‐H qPCR negative; black horse, EqPV‐H qPCR positive

Week 19 testing indicated that 8 of the 9 horses had hepatitis. Six of these were serum EqPV‐H qPCR‐positive, including horses RQ, BU, and FO (Figure 2). Additionally, Horse CY was serum EqPV‐H qPCR‐positive at week 19 and developed hepatitis at week 20 (Figure 2). The 4 horses that became viremic between week 12 and 19 after IN inoculation had all seroconverted by week 19 to 20.

Two horses with hepatitis at week 19 were qPCR‐negative (Horses RK and FA; Figure 2). Horse RK developed viremia at week 22 (Figure 2). In total, 8 of the 9 horses were serum EqPV‐H qPCR‐positive by 22 weeks post‐IN inoculation (Figure 1A). Horse FA did not become viremic by 23 weeks after IN inoculation, and later was confirmed to be susceptible to EqPV‐H by IV inoculation (Figures 1A and 2).

### 3.5 Group B inoculations and results

As with group A, group B (n = 2) horses were inoculated first PO and then IN, and monitored as described above. The horses neither became viremic nor seroconverted at 8 weeks after PO inoculation on April 5th (Figure 1B). However, they did develop viremia 6 and 10 weeks after IN inoculation (Figure 1B), which fits within the time frame of becoming EqPV‐H PCR positive after IN inoculation observed in the horses from group A (Figure 1A). One of the 2 horses developed hepatitis concurrent with viral clearance, and the other 1 never developed hepatitis.

### 3.6 Viral kinetics and serology

**FIGURE 3:**
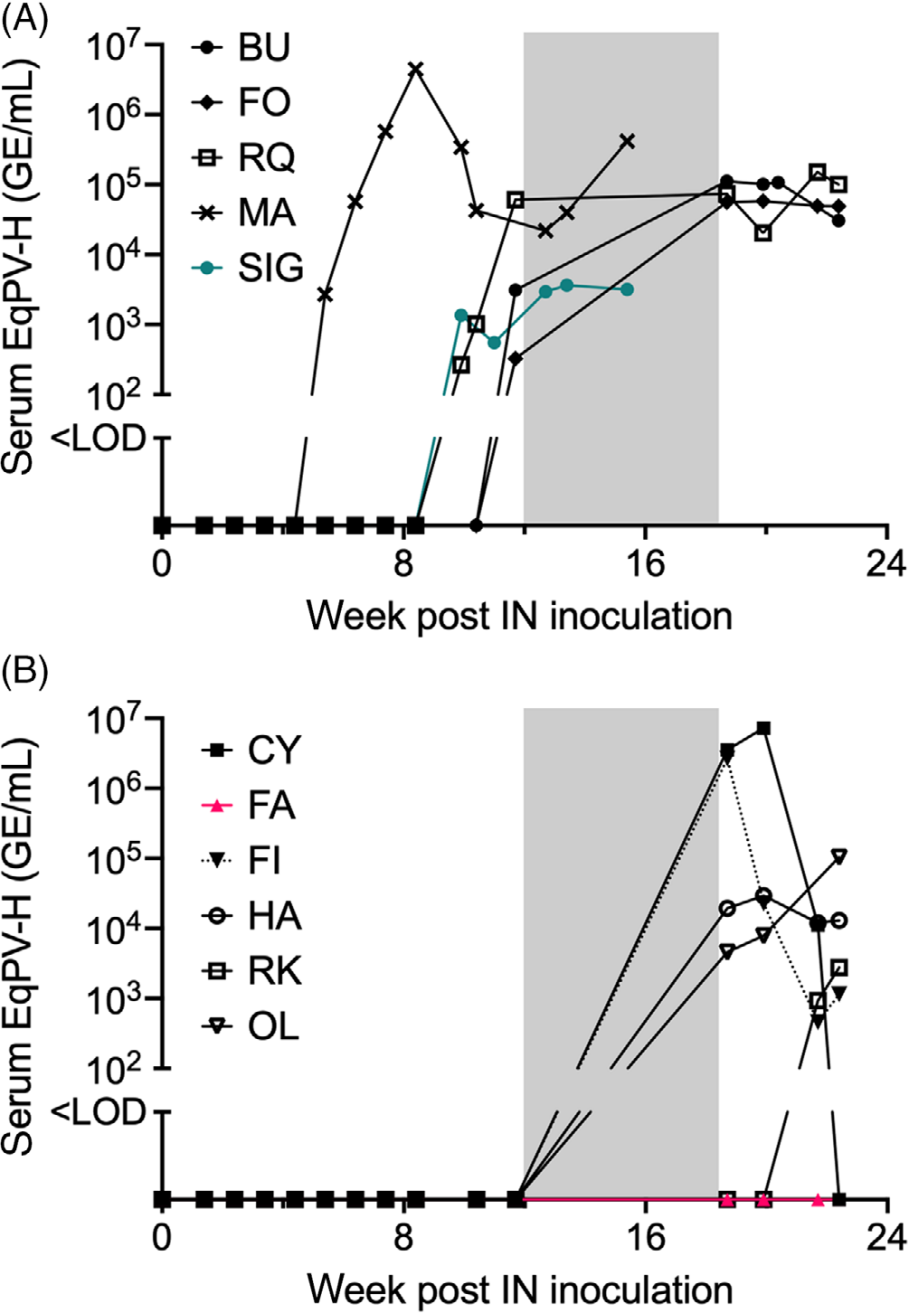
EqPV‐H viremia after intranasal (IN) inoculation. (A) Five horses became viremic within 12 weeks after IN inoculation. All but one (SIG, aqua) developed hepatitis. (B) Four horses became viremic sometime between 12 and 19 weeks, while monitoring was suspended. A fifth horse became viremic at 22 weeks. All 5 had hepatitis. One horse (FA, pink) never became infected and was later shown to be susceptible to EqPV‐H by IV inoculation. Gray shaded boxes correspond to the 7‐week period without monitoring of group A because of herd illness

**FIGURE 4:**
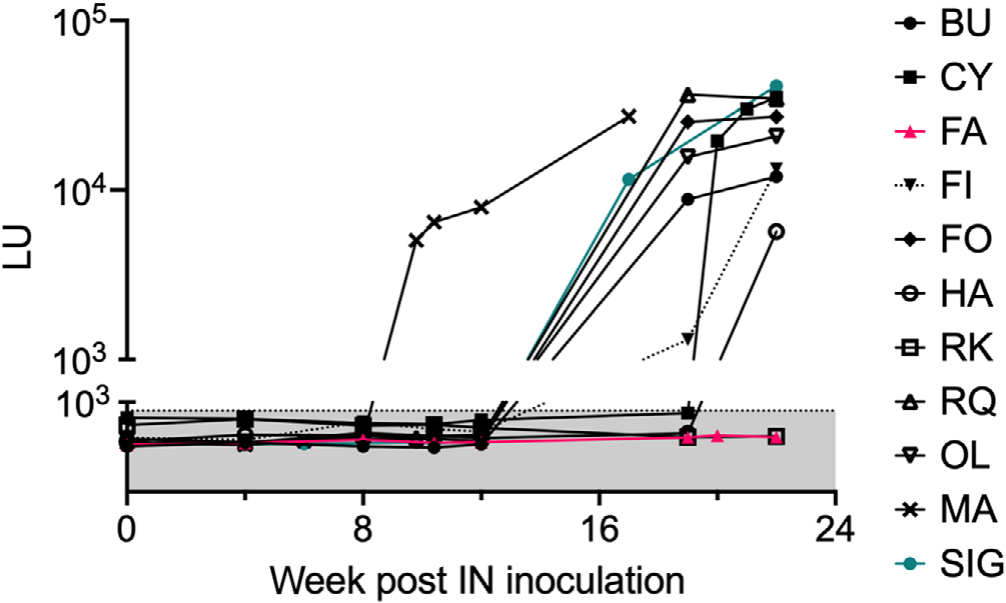
Seroconversion after intranasal (IN) inoculation. Antiviral protein 1 immunoglobulin G antibodies were detected in serum by LIPS assay. All but 1 horse (FA, pink) seroconverted. One horse (RK) did not seroconvert within 22 weeks, but did seroconvert by 45 weeks (not shown). One horse (SIG, aqua) seroconverted and cleared the infection without detectable hepatitis. Gray shading is below the positive cutoff. LU, light units

Collectively, 5 of the 11 horses (45%) became viremic within 12 weeks after IN inoculation with EqPV‐H, at a median of 10 weeks (range, 6‐12; Figure 3A). An additional 5 horses became viremic between 12 and 22 weeks (Figure 3B). Infection was further confirmed by serology. At 19 weeks post‐IN inoculation, 7 horses already had seroconverted, including the 5 that were viremic before week 12 and 2 that were detected to be EqPV‐H serum PCR positive at week 19 (Figure 4). Two more horses seroconverted at weeks 20 and 22 (Figure 4). Horse RK, which became viremic at 22 weeks, seroconverted sometime later between 33 and 45 weeks after IN inoculation (data not shown).

## 4 DISCUSSION

Our study expands understanding of the transmission modes of EqPV‐H. Iatrogenic transmission of this virus by administration of equine origin blood products has been well documented.^16^ Additional methods of natural horizontal or vertical transmission must occur as well, because EqPV‐H is transmitted among horses that have not received such treatments.^3^ Oral, nasal, and fecal shedding during acute EqPV‐H infection has been demonstrated,^1^ indicating inhalation or ingestion as plausible transmission routes. Here, we observed nasal transmission and found no evidence of PO transmission, which contradicts our previous study where we reported PO but not intranasal transmission in 1 of 2 horses.^1^ In that study, we first inoculated both horses IN, and after a follow‐up period of 8 weeks during which both horses did not become viremic, we proceeded with PO inoculation. Based on our present finding of a prolonged eclipse phase before detectable viremia after nasal transmission, we propose that the 1 horse that became infected 4 weeks after PO inoculation actually was infected from the IN inoculation that occurred 12 weeks earlier.

For group B and the 3 horses in group A that became viremic by week 12, the route of transmission is interpreted to be the experimental nasal inoculation. For the 5 horses that became viremic after week 12, the route of transmission is unclear. Four of these horses were viremic by week 19 and seropositive by week 20. Based on a previously determined median time from onset of viremia to seroconversion of 4 (2.4‐5.4) weeks,^1^ these 4 horses likely became viremic between weeks 14 and 18 after IN inoculation. However, they also were exposed to viremic horses RQ, BU, and FO since weeks 10 to 12. Because horses are shedding virus at the onset of viremia,^1^ this would allow approximately 4 to 8 weeks incubation, if they were infected by horizontal transmission from their herd mates, rather than from the experimental IN inoculation itself. Although nasal, PO, and fecal shedding has been reported in acutely infected horses,^1^ no samples were obtained in our present study to demonstrate shedding from the horses during the rest period. Transmission by biting insects was unlikely. The IN transmission spanned February 4th to June 16th. The spring was fairly cold and there was approximately 1 week of mosquito and tick activity in mid‐April. Fly activity was not observed until approximately June 1st. The first transmission of West Nile Virus in New York state, an indication of mosquito vectoring, was June 18th that year. In summary, we can only conclude that 5 horses were infected by the experimental inoculation, and the route of infection for the remaining 5 horses is undetermined, but likely represents natural horizontal transmission, emphasizing the efficiency of transmission within a herd.

A limitation of our study is the small number of horses, combined with the unexpectedly prolonged eclipse phase and unintended cohousing of viremic horses with nonviremic horses during the rest period. This situation allows us to conclude that EqPV‐H can be transmitted nasally but precludes evaluation of transmission efficiency. Additionally, concurrent diseases in the herd complicated assessment. The respiratory disease could have weakened upper respiratory antiviral defenses and allowed EqPV‐H infection to occur, particularly in the horses that became viremic after 12 weeks post‐IN inoculation. Similarly, at least 1 horse, FA, had hepatitis at week 19 without EqPV‐H infection, allowing for the possibility that some other hepatic insult could have promoted EqPV‐H infection in the herd. One hypothesis of how EqPV‐H replicates in hepatocytes, a typically nondividing cell population, is the “two‐hit” theory stating that concurrent hepatic damage either causes hepatocyte replication or upregulates DNA damage repair pathways, resulting in the activation of host DNA polymerases that can promote viral genome replication. These factors could have increased transmissibility of EqPV‐H in this group. However, a quite high herd prevalence of up to 60% to 100% occurs on farms with Theiler's disease,^3^, ^27^ suggesting the transmission observed in our study would not be unreasonably high.

Successful transmission of a virus with experimental inoculations, such as performed here, does not always correspond to efficient transmission in nature. The dose utilized was 5 million GE, which is reasonable because nasal shedding has been documented with up to 1 million GE in a single swab.^1^ However, most shedding was observed at much lower levels, and thus it remains unclear what viral doses horses actually are exposed to in nature, as well as the required minimum infectious dose of EqPV‐H for a horse to become infected. Additionally, spontaneous cases of Theiler's disease (the severe outcome of EqPV‐H infection) show a seasonal distribution in the summer to fall.^3^ Whether this observation indicates seasonal transmission that is restricted to spring and summer based on the presence of insects, or whether other seasonal factors contribute to this severe manifestation of disease remains unknown. Given that parvoviruses are not known to be biologically vectored by insects, insect transmission would have to be mechanical either by face and house flies transferring externally shed virus or by biting flies, such as horse and stable flies, transferring blood. In our present study, little (if any) fly activity was documented during the time period of this experimental transmission study, further supporting the concept of nasal transmission.

The finding that transmission can occur by nasal exposure has important implications for biosecurity and vaccine design. Ideally, isolation of EqPV‐H‐infected individuals could be utilized to prevent transmission on a premise. However, doing so presents multiple challenges. First, EqPV‐H shedding has been documented for at least 10 weeks after acute infection.^1^ Second, horses can be chronically infected for years,^16^, ^24^ and shedding during chronic infection has not yet been evaluated. Third, our study demonstrated a prolonged incubation period of up to 12 weeks before detectable viremia. Thus, both isolation of infected horses and monitoring of in‐contact horses would require separation and repeated sampling, respectively, for many weeks or months. Vaccination provides an attractive alternative when an effective biosecurity approach to limit viral spread is impractical. Because many effective vaccines are available for various parvoviruses, it appears feasible also to develop an effective vaccine for EqPV‐H. Based on our findings of transmission via nasal mucosal surfaces, we propose that a vaccine inducing robust local immunoglobulin A responses in the nose could be highly effective.^35^

## CONFLICT OF INTEREST DECLARATION

The content is solely the responsibility of the authors and does not represent the official views of the National Institutes of Health.

## OFF‐LABEL ANTIMICROBIAL DECLARATION

Authors declare no off‐label use of antimicrobials.

## INSTITUTIONAL ANIMAL CARE AND USE COMMITTEE (IACUC) OR OTHER APPROVAL DECLARATION

All procedures were approved by Cornell University IACUC, #2014‐0024.

## HUMAN ETHICS APPROVAL DECLARATION

Authors declare human ethics approval was not needed for this study.
